# Treatment-resistant depression: time to rethink current definitions and clinical practice

**DOI:** 10.3389/fpsyt.2025.1733678

**Published:** 2026-01-14

**Authors:** Walter Paganin

**Affiliations:** Studio Psicologia Signorini, Guidonia, Lazio, Italy

**Keywords:** treatment-resistant depression, difficult-to-treat depression, personalized medicine, clinical management, biopsychosocial approach to depression

## Introduction

### The legacy framework of TRD

Treatment-resistant depression (TRD) is a construct that was first introduced in the 1970s (1), and was further developed in subsequent decades through staging models such as those proposed by Thase and Rush (1997) and the Maudsley Staging Method (2009), offering useful frameworks that nonetheless failed to resolve persistent definitional heterogeneity (2). Despite nearly thirty years of scientific progress, continued reliance on conceptual models elaborated in an earlier phase of psychiatry limits the integration of contemporary evidence into both the definition and clinical management of TRD. The ongoing lack of consensus on what constitutes “treatment resistance” reveals underlying conceptual limitations (3, 4); and underscores the need for a critical re-evaluation of the construct in light of current evidence, together with the development of updated conceptual frameworks that integrate recent psychiatric advances and move toward a more unified and operational definition (4). The systematic review by Brown et al. (2019) exemplifies this definitional complexity, identifying as many as 155 distinct definitions of TRD across the literature (5). Such variability ranging from the failure of a single antidepressant to multiple unsuccessful trials across different pharmacological classes, undermines study comparability, generalisability of findings, and patient identification for advanced therapeutic strategies. Regulatory authorities have likewise adopted differing operational criteria, with direct implications for treatment access and the comparability of study data. The European Medicines Agency (EMA), in its *Guideline on the Clinical Investigation of Medicinal Products in the Treatment of Depression* (EMA/CHMP/185423/2010 Rev. 3, 2025), drawing on the model proposed by Souery (1999), defines TRD as a lack of clinically meaningful improvement following two antidepressant trials from different pharmacological classes, each administered at an adequate dose and duration with documented treatment adherence (6). Unlike the quantitative symptom reduction thresholds derived from Delphi consensus methods (<25% reduction on the Hamilton Depression Rating Scale or Montgomery–Åsberg Depression Rating Scale for non-response, and 25–50% for partial response) (7), the EMA guidelines do not specify numerical response thresholds. The U.S. Food and Drug Administration (FDA) requires more stringent documentation, namely the failure of two oral antidepressants at therapeutic doses for 6–8 weeks with verified adherence (8). The lack of harmonised criteria across major guidelines (2018–2019) continues to allow methodological variability that complicates the interpretation and synthesis of study results. Differences in regulatory definitions contribute to maintaining clinical and methodological variability, complicating cross-study comparison and limiting the reliability of meta-analytical approaches. This heterogeneity has emerged alongside the development of pharmacological agents specifically targeting TRD and reflects the challenge of balancing regulatory requirements, research objectives, and clinical applicability. Moreover, the current emphasis on pharmacological criteria may inadvertently underrepresent psychosocial and functional dimensions that are essential for a comprehensive assessment of therapeutic response. Recent literature advocates integrating psychological, functional, and contextual factors, moving beyond a purely pharmacological model (4) (3).

In light of these limitations, several authors have proposed reconceptualising TRD as part of a broader continuum of therapeutic difficulty, incorporating cases of difficult-to-treat depression (DTD) in which significant symptoms and functional impairment persist despite appropriate therapeutic efforts (9, 10). This dimensional perspective, consistent with the principles of personalised medicine, seeks to transcend the dichotomous “responder/non-responder” framework and to foster a more nuanced understanding of the factors that shape treatment outcomes.

## Discussion

### 155 definitions, 0 consensus and industry influence

The 155 definitions of treatment-resistant depression (TRD) catalogued by Brown and colleagues in 2019 diverge on almost every parameter, ranging from the number of required therapeutic failures (from one to ≥5), to minimum trial duration (≈2 to ≥12 weeks), dose thresholds (“minimum effective” vs “maximum tolerated”), response/nonresponse criteria, and whether psychotherapy and/or neuromodulation are included, with concrete effects on clinical eligibility and the comparability of studies. This variability means the same individual may qualify as TRD in one setting but not another, with repercussions for access to advanced treatments, outcome interpretation, and reimbursement policies (5). Broader definitions tend to enlarge the eligible population, with inevitable economic implications for health systems (particularly for high-cost interventions), without per se guaranteeing improved clinical value (11). The scientific literature has become so heterogeneous that a meaningful meta-analysis is often infeasible. A further, worrisome issue is pseudoresistance. While 30-60% of patients show incomplete response, many reflect pseudoresistance from suboptimal treatment rather than true TRD (12), that is, apparent failure driven by inadequate dosing, poor adherence, nontherapeutic dose intensity, or inadequately recognized diagnosis/comorbidities, which can artificially inflate “resistance” rates when trial adequacy is not rigorously documented (dose, duration, adherence; sometimes plasma levels) (13, 14);. Compounding this is ambiguity about what constitutes an “adequate dose,” particularly in the presence of wide interindividual variability: for CYP substrates, metabolic differences of roughly an order of magnitude (≈10×) have been documented, rendering standard doses inappropriate for non-negligible subgroups (15). Finally, the current definitional framework reduces the phenomenon to pharmacology, underestimating decades of evidence for the efficacy of psychotherapies (CBT, IPT, and others) (16) and neglecting key individual determinants, trauma, social support, concurrent stressors, and medical comorbidities, that shape treatment response and outcomes. The haste to declare resistance, driven by algorithmic protocols, precludes adequate treatment optimization. The result is a construct poorly aligned with the principles of personalized medicine, with clinical consequences (premature “resistance” labeling; pseudoresistance due to inadequate dose/duration/adherence) and economic consequences (equitable, value-based allocation) that demand more rigorous, multidimensional operational criteria. Overall, the TRD “definitional chaos” reflects a misalignment among clinical needs, regulatory practices, and research uses: while pragmatic criteria facilitate trial enrollment and the authorization of new therapies, they compromise study comparability, generate inter-center decision variability, and obscure key domains such as functioning and quality of life (11, 17). Consequently, both research and clinical practice should make explicit and transparent operational criteria (number of trials, dose and duration, adherence, patient-relevant outcomes), adopt dimensional frameworks (e.g., staging, PRD/TRD profiles), and systematically assess economic impact and sustainability (11). Consistent with this need, Hannah et al. (2023) reviewed 31 economic evaluations and highlighted substantial methodological heterogeneity and the need for more rigorous models, reinforcing the urgency of early differentiating criteria (DTD vs TRD) and economically sustainable intervention strategies (18).

### Deconstructing TRD’s practical benefits

The TRD construct has undoubtedly yielded practical benefits: it has supplied clinicians with a framework to identify patients who may require alternative therapeutic approaches, facilitated insurance coverage for specialized interventions, and created a research category for the study of hard-to-treat populations. However, pronounced operational heterogeneity across studies and guidelines reduces comparability, undermines interpretability, and constrains generalizability and clinical translatability, resulting in considerable conceptual inconsistency (7, 11, 19);

### Clinical consequences: iatrogenesis and reductionism

The TRD framework may lead to what Fava and Rafanelli (20) describe as “*cascade iatrogenesis*”. Algorithmic protocols such as the Texas and German Medication Algorithm Projects (TMAP) and (GAP), have improved standardisation but can promote repetitive pharmacological cycling before addressing psychosocial barriers, adherence, or neuromodulation. Effective management requires multidimensional algorithms with periodic re-evaluation of treatment adequacy, ensuring that interventions target overall quality, adherence, and patient-centred outcomes. Pseudoresistance, apparent non-response due to inadequate dose, duration, adherence, or diagnostic accuracy rather than biological refractoriness, represents a major source of misclassification (12–14). Evidence shows that suboptimal adherence and trial inadequacy explain many so-called resistant cases, with non-adherence rates in major depression ranging from 30% to 60% (21–24). Structured verification of adequacy, including ≥6–8 weeks at therapeutic dose, adherence assessment, and, when appropriate, therapeutic drug monitoring (TDM), can reduce misclassification and improve outcomes (25). Pharmacogenomic variability also contributes to apparent resistance. CYP2D6, CYP2C19, and CYP2B6 polymorphisms may produce up to tenfold differences in antidepressant exposure, influencing efficacy and tolerability. CPIC 2023 guidelines recommend dose or drug adjustments for poor and ultrarapid metabolisers (26). Integrating pharmacogenomic data with TDM and clinical evaluation helps differentiate true resistance from pharmacokinetic mismatch. Polypharmacy remains a systemic concern: many patients receive multiple psychotropics without proven synergistic benefit, increasing adverse effects and treatment burden (27).

### Staging models: complexity without clarity

**Figure 1:**
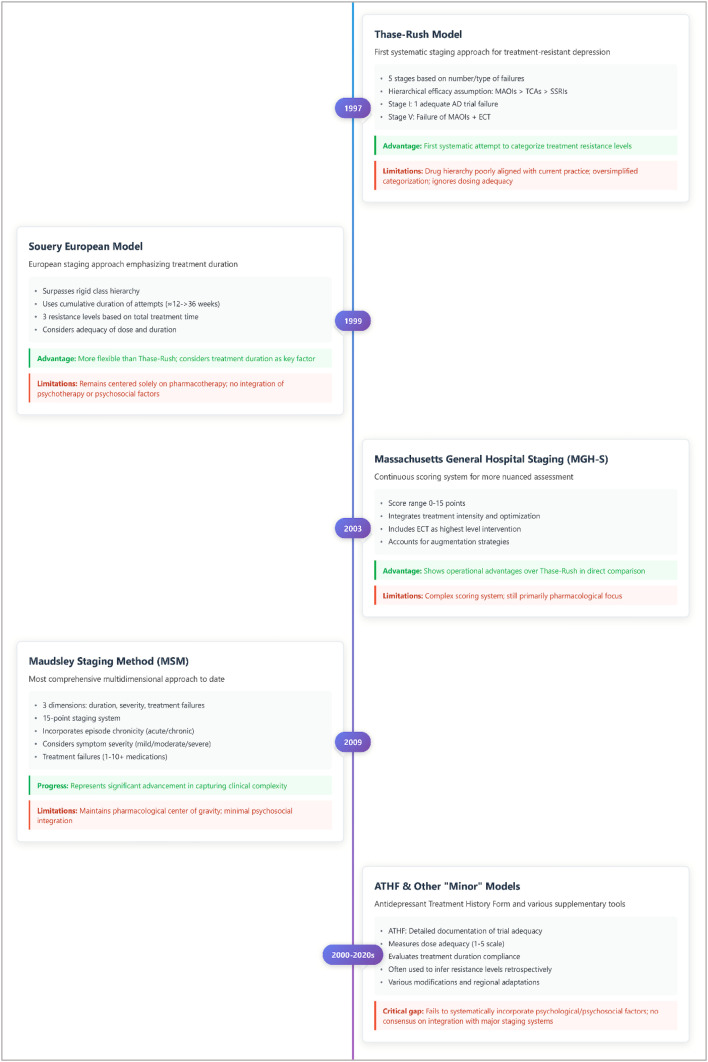
Evolution of TRD staging models (1997-2020s).

Since the 1990s, numerous staging systems have sought to quantify “resistance,” yet none has achieved external cross-validation or international consensus. As **Figure 1** illustrates, escalating methodological complexity ranging from early hierarchical frameworks to institutional staging systems and treatment-history, based instruments, has not resolved definitional heterogeneity or the underlying pharmacocentric bias, while increasing operational complexity without clear, shared predictive value (28). These models typically combine the number of failed antidepressant trials, treatment duration, and comorbidities into cumulative scores to stratify patients by severity or likelihood of non-response. Comparative studies reveal low concordance across systems; for example, overlap between the Maudsley and Thase–Rush classifications for “severe TRD” is only 60–70%, with limited predictive validity for functional or quality-of-life outcomes (3, 19, 28, 29), Most frameworks remain pharmacocentric, overlooking adherence, psychotherapy, and psychosocial determinants. Emerging dimensional approaches, such as the DTD model, propose a broader integration of biological, psychological, and contextual factors, prioritising functional recovery. Staging models should therefore be viewed as useful but limited heuristic tools: they offer structured classification but lack validation across diverse populations and fail to capture the multidimensional nature of chronic depression. Future work should aim to develop hybrid systems integrating pharmacological, functional, and psychosocial domains, validated against longitudinal outcomes of recovery and quality of life (17).

### Functional outcomes and recovery

Beyond symptom reduction, functional recovery has become a central outcome in TRD management. Instruments such as the *Sheehan Disability Scale (SDS)*, *WHODAS 2.0*, and *Work and Social Adjustment Scale (WSAS)* assess functioning across occupational, social, and family domains (17). Functional recovery is generally defined as SDS ≤12 or WSAS ≤10, with *SDS 13–20* indicating partial recovery (9, 29). T These cut-offs align with *functional response (SDS ≤12)* and *remission (SDS ≤6)* criteria proposed by Kennedy (2022), emphasising that recovery often extends beyond symptom remission (30). Even patients meeting remission thresholds (MADRS <10, HAM-D <7) frequently show residual functional disability, underscoring the need for combined symptom- and function-based assessment consistent with DTD consensus recommendations (9).

### Beyond TRD: the DTD framework

Given these fundamental limitations of the TRD paradigm, definitional chaos, iatrogenic consequences, and pharmacocentric reductionism, a reconceptualization is urgently needed. Shifting the focus from counting pharmacological failures to a dimensional assessment would appear to be more operationally useful. Instead of TRD, a true paradigm shift is needed, one that highlights the clinical complexity and multidetermined nature of the treatment difficulty. Difficult-to-Treat Depression refers to a condition in which depression continues to produce a significant clinical and functional burden despite usual therapeutic efforts. This assessment integrates not only the adequacy of treatment trials (dose, duration, adherence) but also symptomatic patterns, functional impairment, psychiatric and medical comorbidities, psychosocial stressors, patient preferences and goals, as well as organizational barriers such as difficulties in accessing services and care continuity (31, 32). DTD moves beyond the binary resistance/non-resistance logic typical of TRD and recognizes a spectrum of response (remission, partial response, nonresponse), orienting practice toward a disease-management approach that aims to optimize functioning and quality of life in addition to symptom reduction (9, 10). Fundamentally, DTD frameworks integrate early psychotherapeutic and social interventions (CBT/CBASP, mindfulness-based approaches, multifamily therapies, rehabilitation and social support) and neuromodulation when indicated, thus avoiding the late deployment of these resources after prolonged sequences of pharmacological switches and augmentation strategies. Organizationally, DTD implies collaborative-care models (psychiatrist, psychologist, social worker, GP) with periodic reassessment of diagnosis and treatment adequacy, shared decision-making, and family involvement (10). On this basis, supported by international consensus/roadmaps, DTD can serve as a platform for study and service design capable of overcoming TRD’s definitional heterogeneity (9).

### Integrated biopsychosocial approaches

Effective management of TRD requires an integrated, multimodal strategy addressing biological, psychological, and social determinants of health. Evidence supports early use of psychotherapies such as CBASP, mindfulness-based cognitive therapy (MBCT), and Schema Therapy, rather than reserving them for pharmacological failures. Multifamily Therapy (MFT) also shows benefit in resistant and difficult-to-treat depression by improving social and emotional functioning and involving families (33), and can be combined with individual psychotherapy and neuromodulation (rTMS/dTMS) for severe cases. Comprehensive care must also target social determinants housing, employment, relationships, trauma/through coordinated collaboration among primary care, psychiatry, psychology, and social services. Digital health tools could further improve management through therapeutic apps, wearable monitoring, and artificial intelligence-based personalization. Within the DTD framework, these resources are deployed early and collaboratively, contrasting with the delayed, sequential pharmacological strategies typical of traditional TRD paradigms.

## Conclusion

Overall, the current TRD framework, shaped by pharmacocentric bias, persistent definitional heterogeneity, limited integration of functional outcomes, remains conceptually and operationally inadequate and translates into cascade iatrogenesis, polypharmacy, pseudoresistance, delayed access to effective care, inflated healthcare costs, and hindered clinical progress in chronic depressive illness. Emerging dimensional approaches, such as the DTD construct, offer a more comprehensive understanding by incorporating biological, psychological, and contextual determinants. Moving toward this integrated model represents a clinical and ethical priority aimed at improving accuracy in diagnosis, personalisation of care, and patient recovery. Strengthening methodological rigor, harmonising regulatory definitions, and embedding functional and psychosocial metrics into research and practice are essential steps toward a more coherent and clinically meaningful classification of resistant depression.
